# Comparative analysis on HIV patient survival in Arad county

**DOI:** 10.1186/1471-2334-14-S4-P19

**Published:** 2014-05-29

**Authors:** Laura Nicolescu, Dana Negru, Teodora Olaru, Mirandolina Prişcă

**Affiliations:** 1 Public Health Department, Arad, Romania; 2 Arad County Emergency Hospital, Arad, Romania

## 

Survival for HIV patients has considerably improved after the introduction of antiretroviral therapy, viral resistance, co-infections and intercurrent infections monitoring.

We have analyzed HIV registered cases between 1990-2013, starting from the hypothesis that the median survival of patients in the absence of antiretroviral therapy could be only five years, for the first decade of the period under review. Data were analyzed with SPSS.14.0 for Windows and MedCalc.

HIV infection in Arad recorded an incidence comparable to the national average, with a ratio of 2.62 deaths for cases diagnosed in 1990-2000 compared with the period 2001-2013, with death rates of 33% for the first period and 18% for the second. The trend of new cases is slightly decreasing but the death rates remain high. Using methods for risk of death or disease progression measuring, Kaplan-Meier Survival Curve indicates a 4 years (P <0.0001) median survival for HIV patients diagnosed between 1990-2000, with a statistically significant distribution of deaths in males (P = 0.0066), with relative risk of death for 1990-2000 decade compared to 2001-2013, ranging from 1.5464 (95% CI 1.094 to 2.1859). It still remains an increased risk of death for males versus females 1.3065 (P = 0.0125 95% CI 0.9391 to 1.8175).

Antiretroviral therapy reduced the relative risk of death in HIV patients, death rates which are still increased for males.

